# Effective treatment of advanced lung adenocarcinoma with paraneoplastic leukemoid reaction with Lorlatinib: a case report

**DOI:** 10.3389/fonc.2024.1341233

**Published:** 2024-01-26

**Authors:** Ruiqi Niu, Yiruo Zhang, Jingdan Pang, Qing Zhou, Yu Lei, Yingying Du

**Affiliations:** ^1^ Department of Oncology, First Affiliated Hospital of Anhui Medical University, Hefei, China; ^2^ Guangdong Lung Cancer Institute, Guangdong Provincial People’s Hospital (Guangdong Academy of Medical Sciences), Southern Medical University, Guangzhou, China

**Keywords:** non-small cell lung cancer, paraneoplastic syndrome, leukocytosis, Lorlatinib, targeted therapy

## Abstract

**Background:**

Lorlatinib is a new generation ALK kinase inhibitor. We describe a 52-year-old patient with ALK-positive advanced lung adenocarcinoma who achieved remission after multi-line therapy combined with paraneoplastic leukemoid reaction treated with Lorlatinib.

**Case report:**

A 52-year-old male patient was diagnosed with stage IV right lung adenocarcinoma, ALK: (+), previously received oral Crizotinib and Alectinib. Blood routine showed white blood cells abnormally elevated after disease progression, and maximum white blood cell count was 179.14×10^9/L. The patient was enrolled in study entitled “a phase II, multicenter, open-label, dual-cohort study to evaluate the efficacy and safety of LORLATINIB monotherapy in ALK inhibitor-treated locally advanced or metastatic ALK-positive non-small cell lung cancer patients in China”. With oral Lorlatinib, the white blood cell count decreased from 179.14×10^9/L to normal after two weeks of administration. PFS was 4.5 months. When follow up imaging showed lesions progression, the white blood cell count increased again, diagnosing a paraneoplastic leukemic reaction. OS was 5.2 months.

**Conclusion:**

In this case, fourth-line Lorlatinib treatment is effiective in ALK-positive advanced patient with paraneoplastic leukemoid reaction. ClinicalTrials.gov Identifier: NCT03909971

## 1 Introduction

In 1977, PLR was first reported in a patient with lung cancer (1). A white blood cell count of more than 50x10^9/L is termed as a paraneoplastic leukemic reaction(PLR), excluding myeloid neoplastic infiltration in solid tumors, the most common of which is non-small cell lung cancer (2).

It is a case report of an advanced ALK-positive lung adenocarcinoma patient with paraneoplastic leukemoid reaction achieved remission after fourth-line Lorlatinib treatment. We present the following case in accordance with the CARE reporting checklist.

## 2 Case presentation

**Figure 1:**
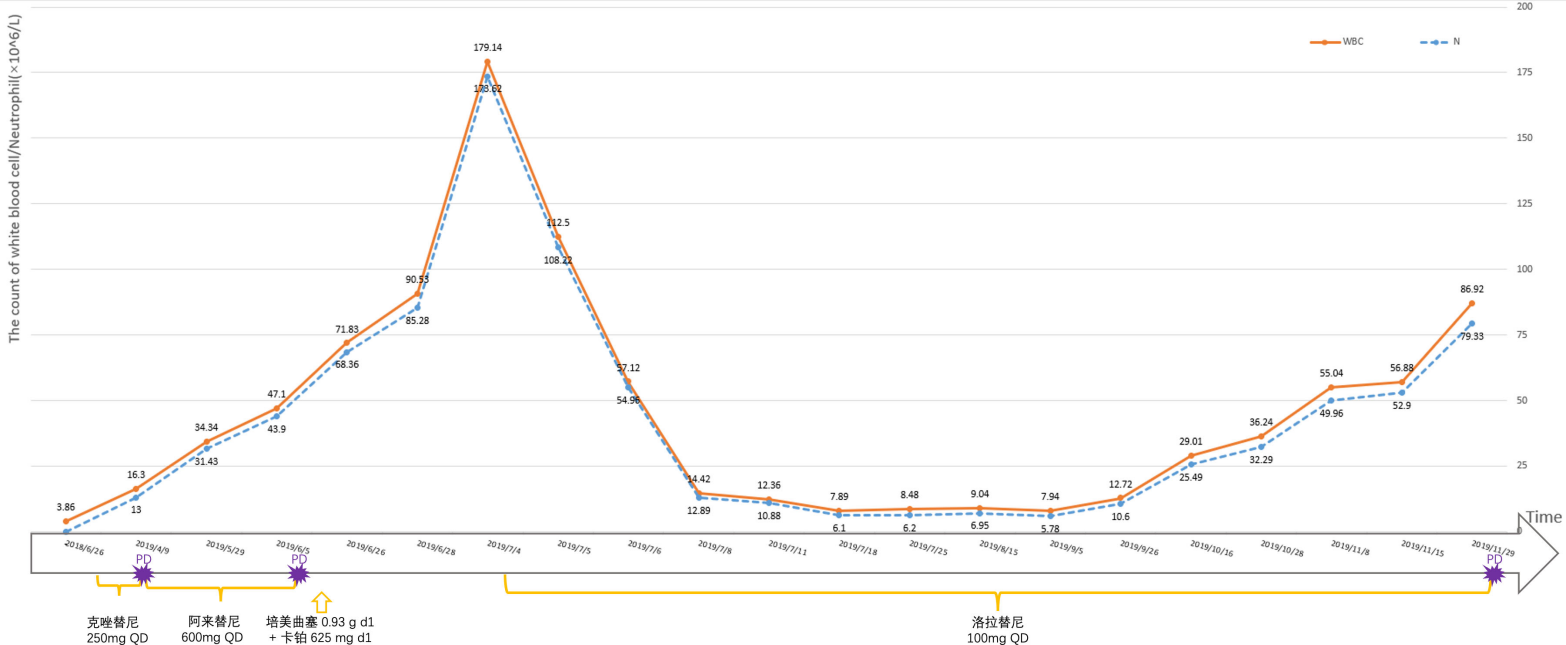
The amount of white blood cells and the number of neutrophils in patients are monitored at different times.

**Figure 2:**
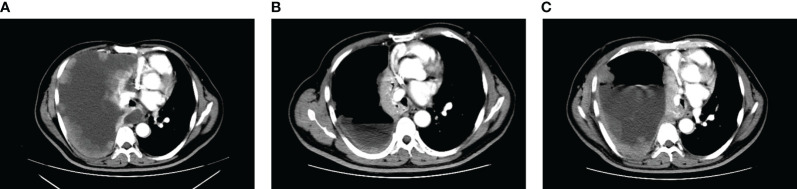
CT images corresponding to the lung lesions in patient before oral Lorlatinib **(A)**, significant remission of pleural lesions and pleural effusion during oral Lorlatinib **(B)**, and pleural lesions and pleural effusion that developed after 5 months of oral Lorlatinib **(C)**.

A 52-year-old male patient was diagnosed with stage IV lung carcinoma in 2018. Lung biopsy, pathology showed adenocarcinoma, ALK-Tissue (+), TTF- 1 (+). The patient has no family history or history of chest disease or psychological disorders. Genetic test results: There was t(2p23) ALK (+) by FISH, with ALK gene translocation. The patient then received oral Crizotinib, during which best overall response(BOR) was SD. PFS was 13.3 months. Treatment was altered to oral alectinib. PFS was 1.7 months. The patient received one course of chemotherapy (pemetrexed plus carboplatin) after the progression of the disease. The count of white blood cells was found abnormally increased two-weeks later, the bone marrow biopsy showed bone marrow tissue, active proliferation of hematopoietic cells, all three lines of hematopoietic cells were visible, without evidence of malignancy. One week later, the patient was treated with thoracentesis drainage for “chest tightness and shortness of breath for 1 month”. The patient had obvious symptoms of chest tightness and shortness of breath, with chest pain, along with dyspnea and sweating. The patient was treated with antiasthmatic and diuretic therapy to alleviate symptoms. No abnormalities were seen in the bone marrow aspiration and biopsy, and the patient’s temperature was normal. The results of procalcitonin and c-reactive protein were under normal range at an outside hospital. Physical examination showed low respiratory sounds in the right lung and dry rales in the left lung. The patient had results of Tumor Abnormal Protein and HSP90 tests at our hospital. The serum Tumor Abnormal Protein was abnormal and has small coagulants. The serum HSP90 level was 132.35 ng/ml (range, 0-82 ng/ml). The patient was enrolled in the “a phase II, multicenter, open-label, dual-cohort study to evaluate the efficacy and safety of LORLATINIB ·monotherapy in ALK inhibitor-treated locally advanced or metastatic ALK-positive non-small cell lung cancer patients in China” with signing informed consent after a week of hospitalization. Maximum white blood cell count was 179.14×10^9/L, considering leukemoid reaction of lung cancer. The amount of white blood cells and the number of neutrophils in patients are presented in **Table 1** and **Figure 1**. In the patient’s laboratory examination, we found a transient elevation of serum calcium and serum corrected calcium. Serum calcium, serum albumin and corrected calcium levels in patients are presented in **Table 2**. The patient started to take oral LORATINIB. As the patient’s white blood cell count dropped back, the patient’s symptoms significantly ameliorated and he was discharged with medication. The patient’s vital signs and hematology and blood were monitored during outpatient follow-up, during which the white blood cell count was back to normal. The best efficacy evaluation was the PR (**Figure 2**). After more than 4 months of oral Loratinib, the patient’s computed tomography (CT) scan showed significant enlargement of nodules in the right pleura and the presence of metastatic nodules in the left adrenal gland. PFS was 4.5 months. Patient described remarkable improvement in symptoms of chest tightness and generalized malaise. Ahead of this, blood routine showed white blood cells increased again in the third month of oral Loratinib. The patient died of respiratory failure and lung infection.

**Table 1:** The main results of venous blood routine during the course of leukocytosis.

Time	2019-06-05	2019-06-26	2019-07-04	2019-07-05	2019-07-06	2019-07-08
WBC(×109/L)	47.10	71.83	179.14	112.50	57.12	14.。、42
Neutrophil(×109/L)	43.90	68.36	173.62	108.22	54.96	12.89
Lymphocyte(×109/L)	1.60	1.45	1.79	1.98	1.30	1.11
Monocyte(×109/L)	1.20	1.73	3.66	2.20	0.66	0.31
Eosinophil(×109/L)	0.35	0.16	0.00	0.02	0.02	0.08
Basophil(×109/L)	0.09	0.13	0.07	0.08	0.18	0.03
RBC(×1012/L)	3.92	3.36	3.33	3.05	3.06	3.01
Hemoglobin(g/L)	126	111.0	112.0	100.0	98.0	98
Platelet(×109/L)	170	195	237	183	176	162
Reticulocytes×1012/L)	–	0.0659	0.0503	0.0458	0.0456	0.0409

**Table 2:** Serum calcium, serum albumin and corrected calcium levels in patients are monitored at different times.

Time	2019-06-26	2019-07-04	2019-07-05	2019-07-06	2019-07-08
Serum calcium (mmol/L)	2.33	2.63	2.33	2.27	2.22
Serum albumin (g/L)	32.7	34.9	33.6	31.2	30.3
Serum corrected calcium (mmol/L)	2.476	2.732	2.458	2.446	2.414

## 3 Discussion

Paraneoplastic syndrome is a group of signs and symptoms seen in cancer patients not directly related to tumor-related metabolic deficiencies, infections, and side effects of antitumor therapy. Eight percent of cancer patients suffer from paraneoplastic syndrome (3), predominantly in lung cancer patients (4, 5). In a retrospective study, it was found that 33 of 227 lung cancer patients were diagnosed with tumor-related leukocytosis (4). Most of these patients had elevated serum G-CSF, GM-CSF and IL-6. The occurrence of tumor-related leukocytosis appears to be an ominous prognostic sign in patients with lung carcinoma. Cytokines such as serum G-CSF, GM-CSF and IL-6 play a mobilizing role in leukocyte elevation. Tumor-secreted cytokines can induce peripheral leukocytosis by stimulating myelopoiesis in the bone marrow. Clinical differentiation of PLR can be diagnosed by testing serum cytokine levels. A PLR caused by tumor production of G-CSF is most common (6). A PLR is a diagnosis of exclusion. Bone marrow aspiration and peripheral blood smear were performed in an outside hospital after the patient’s leukocyte elevation, the results of which showed no evidence of tumor cell infiltration or leukemia, and the report only mentioned that the hematopoietic cells were actively proliferating in a ratio of about 3:1 with fat, and that hematopoietic cells of all three lineages were visible. Peripheral blood smear results were not reported in detail. The patient did not examine for BCR/ABL fusion gene and the JAK2V617F gene mutation. The results of procalcitonin and c-reactive protein were under normal range at an outside hospital. Therefore, the diagnosis of concurrent infection was excluded. The drug associated hyperleukocytosis was not considered as the patient was not using corticosteroids or vasopressors (7).

Lorlatinib is a selective third-generation anaplastic lymphoma kinase (ALK)/ROS1 tyrosine kinase inhibitor (TKI) with broad ALK mutational coverage and high CNS permeability in advanced ALK-positive non–small-cell lung cancer (8). Clinical study in which patient was enrolled reported results that mPFS of patients in cohort 2 (received one ALK TKI other than crizotinib [ ± prior crizotinib]) is 5.6 months.

In this case, the patient had dyspnea and other clinical symptoms due to leukocytosis at admission. After fourth-line oral administration of Lorlatinib, the white blood cell counts gradually decreased, the patient’s general condition was improved and the disease condition was controlled. Abukhiran et al. (9) reviewed 179 reported cases of PLR in solid tumors and analyzed clinical information. The mean overall survival of these patients was 4 months. On univariate analysis, WBC count was the only variable associated with overall survival (P = 0.03). Patients with moderate (WBC count >40 K-100 K/μL) were at 1.5 times increased risk of death (HR = 1.49) and those with extreme hyperleukocytosis (WBC count >100 K/μL) at twice increased risk of death (HR = 1.99) relative to those with mild leukocytosis (WBC count <40 K/μL). A retrospective study found that survivals of patients with hypercalcemia alone (median survival time(MST) 3.8 months, n = 59), leukocytosis alone (MST 1.9 months, n = 10), and the hypercalcemia-leukocytosis syndrome (MST 1.5 months, n = 6) were significantly shorter than those without them (MST 9.5 months, n = 1074; P < 0.001) (10). Hypercalcemia—leukocytosis syndrome is an indicator for poorer outcome in lung cancer patients. Considered with extreme hyperleukocytosis (WBC count >100 K/μL) and transient hypercalcemia, the patient have poor prognostic factors. At the time of diagnosis of lung cancer, there was no significant abnormality in blood routine and abnormal elevation of white blood cells. Upon tumor progression, the patient gained a paraneoplastic leukemoid reaction (11). Before the radiographic disease progression, the patient’s follow-up blood routine showed significantly increased white blood cells. The temporal changes of white blood cell count in patients further demonstrate that PLR can reflect the control of patients’ tumors and even predict tumor burden and tumor progression trends.

Due to the limited knowledge of ALK-positive lung cancer, leukemoid paraneoplastic syndrome in ALK-positive patients with lung cancer have not been observed. The patient was examined at an outside hospital for bone marrow biopsy, peripheral blood smear, serum tumor markers. When diagnosing the patient’s PLR, When diagnosing the patient’s PLR, there was a lack of testing of serum G-CSF, GM-CSF, and IL-6, making it difficult to diagnose differentiation of PLR. It is the first report that Lorlatinib is effective for ALK-positive NSCLC with PLR.

## Data availability statement

The original contributions presented in the study are included in the article/**Supplementary Material**. Further inquiries can be directed to the corresponding author.

## Ethics statement

The studies involving humans were approved by Clinical Medical Research Ethics Committee of the First Affiliated Hospital of Anhui Medical University. The studies were conducted in accordance with the local legislation and institutional requirements. The participants provided their written informed consent to participate in this study. Written informed consent was obtained from the individual(s) for the publication of any potentially identifiable images or data included in this article.

## Author contributions

RN: Investigation, Writing – original draft. YZ: Resources, Writing – original draft. JP: Resources, Writing – review & editing. QZ: Methodology, Supervision, Writing – review & editing. YL: Investigation, Resources, Validation, Writing – review & editing. YD: Conceptualization, Project administration, Supervision, Writing – review & editing.

## References

[1] Asano S Urabe A Okabe T Sato N Kondo Y. Demonstration of granulopoietic factor(s) in the plasma of nude mice transplanted with a human lung cancer and in the tumor tissue. Blood (1977) 49(5):845–52. 300638

[2] Chakraborty S Keenportz B Woodward S Anderson J Colan D. Paraneoplastic leukemoid reaction in solid tumors. Am J Clin Oncol (2015) 38(3):326–30. doi: 10.1097/COC.0b013e3182a530dd 24145395

[3] Pelosof LC Gerber DE. Paraneoplastic syndromes: An approach to diagnosis and treatment. Mayo Clinic Proc (2010) 85(9). 838–854 10.4065/mcp.2010.0099 PMC2931619 20810794

[4] Kasuga I Makino S Kiyokawa H Katoh H Ebihara Y Ohyashiki K. Tumor-related leukocytosis is linked with poor prognosis in patients with lung carcinoma. Cancer (2001) 92(9):2399–405. doi: 10.1002/1097-0142(20011101)92:9<2399::aid-cncr1588>3.0.co;2-w 11745296

[5] Granger JM Kontoyiannis DP. Etiology and outcome of extreme leukocytosis in 758 nonhematologic cancer patients: A retrospective, single-institution study. Cancer (2009) 115(17):3919–23. doi: 10.1002/cncr.24480 19551882

[6] Kanaji N Watanabe N Kita N Shuji Bandoh S Tadokoro A Ishii T. Paraneoplastic syndromes associated with lung cancer. WJCO (2014) 5(3):197. doi: 10.1016/s0025-6196(12)60050-0 25114839 PMC4127595

[7] Hasjim BJ Grigorian A Stopenski S Swentek L Sun B Livingston JK. Moderate to severe leukocytosis with vasopressor use is associated with increased mortality in trauma patients. J Intensive Care Soc (2022) 23(2):117–23. doi: 10.1177/1751143720975316 PMC9125442 35615240

[8] Shaw AT Solomon BJ Besse B Bauer TM Lin C-C Soo RA. *ALK* resistance mutations and efficacy of lorlatinib in advanced anaplastic lymphoma kinase-positive non–small-cell lung cancer. JCO (2019) 37(16):1370–9. doi: 10.1200/JCO.18.02236 PMC6544460 30892989

[9] Abukhiran I Mott SL Bellizzi AM Boukhar SA. Paraneoplastic leukemoid reaction: case report and review of the literature. Pathol - Res Pract (2021) 217:153295. doi: 10.1016/j.prp.2020.153295 33341546

[10] Hiraki A Ueoka H Takata I Gemba K Bessho A Segawa Y. Hypercalcemia–leukocytosis syndrome associated with lung cancer. Lung Cancer (2004) 43(3):301–7. doi: 10.1016/j.lungcan.2003.09.006 15165088

[11] McCoach CE Rogers JG Dwyre DM Jonas BA. Paraneoplastic leukemoid reaction as a marker of tumor progression in non-small cell lung cancer. Cancer Treat Commun (2015) 4:15–8. doi: 10.1016/j.ctrc.2015.03.003 PMC4410270 25932381

